# Differential Methylation of Genes Associated with Cell Adhesion in Preeclamptic Placentas

**DOI:** 10.1371/journal.pone.0100148

**Published:** 2014-06-25

**Authors:** Lauren Anton, Amy G. Brown, Marisa S. Bartolomei, Michal A. Elovitz

**Affiliations:** 1 Maternal and Child Health Research Program, Department of Obstetrics and Gynecology, Perelman School of Medicine at the University of Pennsylvania, Philadelphia, Pennsylvania, United States of America; 2 Department of Cell & Developmental Biology, Perelman School of Medicine at the University of Pennsylvania, Philadelphia, Pennsylvania, United States of America; VU University Medical Center, Netherlands

## Abstract

Preeclampsia (PE), a hypertensive disorder of pregnancy, is hypothesized to be associated with, if not mechanistically related to abnormal placental function. However, the exact mechanisms regulating the pathogenesis of PE remain unclear. While many studies have investigated changes in gene expression in the PE placenta, the role of epigenetics in PE associated placental dysfunction remains unclear. Using the genome-wide Illumina Infinium Methylation 450 BeadChip array, we analyzed gene-specific alterations in DNA methylation in placental biopsies collected from normal pregnant women delivering at term (n = 14), with term PE (≥37 weeks; n = 19) or with preterm PE (<37 weeks, n = 12). Of the 485,582 gene loci on the array, compared to controls, 229 loci were differentially methylated in PE placentas and 3411 loci were differentially methylated in preterm PE (step up p-value <0.05 and >5% methylation difference). Functional annotation of the differentially methylated genes in preterm PE placentas revealed a 32 gene cluster in the cadherin and cell adhesion functional groups (Benjamini p<0.00001). Hypermethylation of CDH11 (p = 0.0143), COL5A1 (p = 0.0127) and TNF (p = 0.0098) and hypomethylation of NCAM1 (p = 0.0158) was associated with altered mRNA expression in preterm PE placentas. Demethylation of first trimester extravillous trophoblast cells resulted in altered CDH11 (p = 0.0087), COL5A1 (p = 0.0043), NCAM1 (p = 0.0260) and TNF (p = 0.0022) mRNA expression. These studies demonstrate aberrant methylation, correlating with disease severity, in PE placentas. Furthermore, we provide evidence that disruption of gene-specific methylation in preterm PE placentas and first trimester trophoblasts is significantly associated with altered gene expression demonstrating that epigenetic modifications early in pregnancy can have effects on trophoblast function contributing to PE.

## Introduction

Preeclampsia (PE), a hypertensive disorder of pregnancy, is one of the leading causes of maternal and fetal morbidity and mortality worldwide. Affecting 5–10% of pregnancies [1]–[3], PE is an idiopathic disorder characterized primarily by maternal hypertension and proteinuria. PE has a highly varied phenotype ranging from mild increases in blood pressure to a multi-organ system disease that can include seizures, hemolysis, liver and renal injury. The pathogenesis of PE and the mechanisms leading to the different phenotypes of this disease remain unknown. While several theories have suggested genetic, immunologic, placental and endothelial abnormalities contribute to the development of PE, it is generally agreed that the origins of PE lie within the placenta as early delivery and removal of the placenta remain the only cure. The predominant and most widely accepted theory suggests that the pathogenesis of preeclampsia is associated with defective extravillous trophoblast remodeling of the uterine spiral arteries. This defective trophoblast invasion results in decreased vascular flow into the placenta creating a locally hypoxic environment ultimately leading to placental endothelial dysfunction, oxidative stress and increased release of syncytiotrophoblast debris and anti-angiogenic molecules. Abnormalities in the implantation and placentation process, including defective trophoblast invasion and the consequent placental dysfunction, have been shown to contribute to the pathogenesis of PE [4], [5].

In an attempt to help clarify the molecular mechanisms regulating PE associated placental dysfunction, many studies have investigated alterations in gene function and expression within the placenta using large scale microarray-based gene expression profiling [6]–[8]. In a review of 18 microarray based placenta/preeclampsia gene association studies, Louwen, et al. [9] concluded that these studies implicate the involvement of many different placental gene signatures in the development of PE highlighting the complex molecular pathogenesis of this disease. Despite the inconsistencies between the 18 studies, some overlapping placental gene pathways were identified to be associated with PE including trophoblast motility and invasion, angiogenesis, cell survival and immune response. While these gene expression studies have identified many gene targets associated with PE and possibly alterations in placental function, the transcriptional regulation of these genes remains unknown.

Recently, studies have focused on the contribution of placental epigenetic modifications to the development of PE. Epigenetics is defined as both heritable and transient changes in gene expression that do not entail a change in the primary DNA sequence [10]. DNA methylation, the best characterized form of epigenetic modification, is based on a mechanism of methylated cytosines. DNA methylation resulting from environmental insults can be stably transmitted through maintenance DNA methyltransferases (DNMTs) [11], [12]. The placenta, situated at the interface between the mother and fetus, is exposed to a variety of environmental exposures including smoking, nutritional deficiencies, dietary excesses, assisted reproductive technologies and both biobehavioral and molecular equivalents of stress making alterations in placental DNA methylation biologically plausible.

The field of epigenetics is rapidly evolving as an increasing number of complex diseases have recently been shown to be associated with alterations in DNA methylation including diverse types of cancer [13]–[15] and cardiovascular disease [16]–[18] among many others. There has been a significant amount of work investigating the relationship between epigenetics, normal placentation and early embryo development, however, there is a limited amount of data regarding how epigenetic mechanisms may alter placental function and hence promote a disease phenotype such as PE. There are several opinion papers that have suggested that epigenetic mechanisms are involved in PE [19]. Consequently, progress has been made in identifying global methylation profiles in the PE placenta as several studies have performed methylation arrays [20]–[22]. Additionally, there are several studies to date that have investigated alterations in gene specific DNA methylation within the PE placenta including *SERPINA3* [23], [24], *MMP-9* [25], *STOX1* [26] and *Syncytin-1* [27]. However, only one study has demonstrated that methylation of a placental gene (*CYP24A1*) can affect placental function, specifically, regulating the level of active vitamin D at the feto-maternal interface [28].

In this study, we hypothesized that preeclamptic placentas have an altered DNA methylation status resulting in abnormal placental gene expression that may contribute to the development of PE. To test this hypothesis, we identified gene specific changes in DNA methylation in preeclamptic placentas compared to controls. We then assessed if those genes with an altered methylation status resulted in a change in placental gene expression. Further, to determine if the methylation associated gene expression changes seen in third trimester PE placentas were also present in the first trimester, we investigated the expression of these genes before and after demethylation in primary extravillous trophoblast cells.

## Materials and Methods

### Ethics Statement

This study was performed with approval from the University of Pennsylvania Institutional Review Board. Written informed consent was obtained from all study participants prior to sample collection.

### Sample Collection

A prospective case-control study (Preeclampsia: Mechanisms and Consequences) was performed between March 2005 and October 2009 at the Hospital of the University of Pennsylvania. Controls were defined as women without hypertension-related complications that presented for delivery at term (≥37 gestational weeks) (n = 14). Cases (n = 31) were identified according to standard American College of Obstetricians and Gynecologists (ACOG) criteria for diagnosing preeclampsia. Based on these pre-specified criteria, case eligibility was determined at the time of enrollment by the study investigators and not by the treating physician. Immediately after delivery, placental biopsies were taken from cases and controls. After removal of fetal membranes and remaining decidual tissue, four biopsies (one from each “quadrant” of the placenta) were taken from the fetal side of the placenta by a trained research coordinator. The tissues were washed in sterile saline to remove maternal blood and immediately frozen in liquid nitrogen and stored at −80°C until use.

### DNA Extraction

Approximately 25 mg of placental tissue was incubated with proteinase K (Qiagen) and RNase A (Qiagen) at 56°C overnight. Genomic DNA was then extracted from the lysed placental tissue following the manufacture's spin column protocol included with the DNeasy extraction kit (Qiagen).

### Methylation Arrays

Genomic DNA (250 ng) underwent sodium bisulfite conversion according to the manufacture's protocol using the EZ DNA Methylation Kit (Zymo Research Corp, Irvine, CA). The resulting bisulfite converted DNA was hybridized to the Illumina Infinium Human Methylation 450 BeadChip (Illumina, San Diego, CA, USA) (referred to as “methylation 450 array” for the remainder of the manuscript) which provides genome wide coverage containing greater than 450,000 methylation sites per sample. Gene probes are targeted across gene regions with sites in the promoter region, 5′UTR, first exon, gene body, and 3′UTR including CpG islands, island shores, and island shelves (sites flanking island shores), CpG sites outside of CpG islands and miRNA promoter regions among others. Amplification, hybridization, washing, labeling and scanning of the Illumina 450 BeadChip was performed by the Molecular Profiling Core Facility at the University of Pennsylvania following the manufacture's protocols. Methylation levels are quantified by beta (β) values derived from the ratio of intensities between methylated and unmethylated alleles. Average β values reported from each gene probe range from 0 (unmethylated) to 1 (completely methylated). Partek Genomics Suite version 6.5 (Partek, Inc., St. Louis MO) was used for data analysis of the β values including average methylation, fold change, percent methylation and the generation of a “step-up” adjusted p-value which corrects for any false negative results. Principle component analysis (PCA) plots were created based on the methylation 450 array results. The full methylation 450 array dataset was submitted to Gene Expression Omnibus (accession # GSE57767).

### Differential Methylation Analysis

Probe specific methylation differences between placental tissues from cases and controls were analyzed. Any probe with a step-up adjusted p-value greater than 0.50 was excluded from further analysis. In order to identify those genes that were most likely to show a significant difference in biological function, methylation differences were defined as specific gene probes with a change in β values (Δβ) between cases and controls greater than 0.05 (5% methylation difference) in addition to a step-up adjusted p-value less than 0.05. In the comparisons between preterm preeclamptic placentas versus controls, the cut off criteria were further tightened to Δβ values greater than 0.05 and a step-up adjusted p-value less than 0.01 in order to decrease the number of significantly different gene probes between these groups. Gene probes meeting the cut off criteria in each comparison were submitted to the Database for Annotation, Visualization, and Integrated Discovery (DAVID) Bioinformatics Resource version 6.7 (http://david.abcc.ncifcrf.gov) [29], [30] to identify the functional biological pathways associated with those genes showing the largest change in methylation status. A list of all genes present on the methylation 450 array was used as background to prevent any bias in the functional annotation analysis. Genes identified by DAVID as being associated with significant gene functional classifications via the functional annotation tool and/or the Gene Ontology (GO Terms) database were further investigated for changes in mRNA expression.

### Pyrosequencing

Pyrosequencing was performed to confirm the methylation status of four genes of interest identified by the methylation 450 array and downstream bioinformatics analysis. We analyzed the methylation status of cadherin 11 (*CDH11*), collagen type V, alpha 1 (*COL5A1*), neural cell adhesion 1 (*NCAM1*) and tumor necrosis factor (*TNF*) by designing primers (pyromark assay design software) around the gene sequence associated with the specific array probes of interest for each gene (*CDH11*: probe # cg26624576, *COL5A1*: probe # cg14237069, *NCAM1*: probe # cg20857767, *TNF*: # cg04425624). Placental genomic DNA (600 ng) from control (n = 14), term (n = 19) and preterm (n = 12) PE placentas was bisulfite treated using the EpiTect Bisulfite Kit (Qiagen) following the manufacturer's protocol. Utilizing the PyroMark PCR kit (Qiagen), 50 ng of bisulfite treated DNA was used for PCR according to the manufacturer's protocol. Forward and reverse primer sequences for each gene are listed in Table S1. The resulting PCR product was run on an agarose gel to test for the presence of a singular PCR product. 10 µL of the verified biotinylated PCR product was used for each sequencing assay. Sequencing primers for each gene are listed in Table S1. Pyrosequencing was performed using the PyroMark Q96MD system and PyroMark Gold 96 reagent kit (Qiagen) following the manufacturer's protocol. Methylation at the CpG site of interest for each gene was analyzed using Qiagen's Pyro Q- CpG software.

### Luminometric Methylation Assay (LUMA)

Utilizing the LUMA assay, the percentage of global methylation in control (n = 14) versus PE (n = 31) placental genomic DNA was measured using pyrosequencing. The LUMA protocol has been described in detail previously [31]. Briefly, the LUMA assay uses a ratio of DNA cleavage by the methylation sensitive restriction enzyme, HpaII (5 units, New England Biolabs, Ipswich, MA), and the methylation insensitive restriction enzyme, MspI (5 units, New England Biolabs). Enzyme digest reactions (HpaII + EcoRI and MspI + EcoRI) were performed on placental genomic DNA (750 ng per digest) at 37°C for 4 hours. Following digestion, each sample was analyzed by pyrosequencing on the PyroMark Q96D system. Data are presented as % global methylation as determine by the formula 1-[(HpaII/EcoRI)/(MspI/EcoRI)]*100.

### Cell Culture

Primary extravillous trophoblast cells (EVT) were isolated from first trimester villous tissue using a modified protocol originally described by Graham et al. [32], [33] and previously published by our research group [34]–[36]. Briefly, finely minced chorionic villi collected from de-identified elective first trimester pregnancy termination tissues were cultured at 37°C in RPMI 1640 medium containing 20% FBS. EVT cells, which outgrow from attached villous fragments, were separated from villous tissue. The isolated EVT cells were cultured and propagated in RPMI 1640 medium containing 20% FBS. The EVT cells used in our experiments were previously characterized by immunostaining for trophoblast cell markers, cytokeratin-7, 8 and -18 and integrin alpha 1[34], [37], [38]. These results are similar to those obtained by other investigators using the same primary cultures and confirm the purity of the EVT cell preparations [32], [33].

### EVT Cell Treatments

EVTs were plated at 1×10^6^ cells/ml in 10 cm plates and allowed to grow overnight. EVTs were treated with the demethylating agent 5-aza-2′-deoxycytidine (AZA, Sigma-Aldrich, St. Louis, MO) (5 umol/L) (n = 6) or dimethyl sulfoxide (DMSO) vehicle control (n = 6) for 4 days. The media was aspirated and Trizol (Invitrogen, Carlsbad, CA) was added to each well, transferred to tubes and stored at −80°C for RNA extraction.

### RNA Isolation and Quantitative PCR Expression

Total RNA was extracted from placenta tissues (100 mg) and EVTs treated with or without AZA using Trizol (Invitrogen) and cDNA was generated using a high capacity cDNA reverse transcription kit (Applied Biosystems, Foster City, CA). TaqMan Gene Expression Assays for *CDH11*, *COL5A1*, *NCAM1* and *TNF* along with TaqMan Universal PCR Master Mix were purchased from Applied Biosystems (Foster City, CA). QPCR reactions were carried out using equivalent dilutions of each cDNA sample on the Applied Biosystems Model 7900 sequence detector PCR machine. Target mRNA was normalized to the amount of 18S RNA in each sample. The relative abundance of the target of interest was divided by the relative abundance of 18S in each sample to generate a standardized abundance for the target transcript of interest.

### Statistical Analysis

For all experiments, excluding results from the methylation 450 array which were analyzed using methods described above, statistical analyses were performed with GraphPad Prism Software (version 4.0, San Diego, CA, USA). For data that were normally distributed, t-tests or one way analysis of variance (ANOVA) were used. If the data were not normally distributed then the non-parametric Mann-Whitney test was used. Results with p <0.05 were considered statistically significant.

## Results

### Clinical and demographic characteristics of case-control study

Table 1 shows the clinical and demographic characteristics of the case-control study. Placental biopsies were collected from a total of 14 controls and 31 cases. The case group was further stratified into 18 term cases and 13 preterm cases based on gestational age at delivery (for the diagnosis of preeclampsia). Mean gestational age at delivery and mean fetal weight was significantly lower in cases versus controls (p<0.05) and in preterm cases versus term cases (p<0.0001). All other parameters including maternal age, race, body mass index and fetal gender were not statistically different.

**Table 1:** Clinical and demographic characteristics of preeclampsia case-control study.

	Case – Control Analysis		Case – Case Analysis	
	Control (n = 14)	Preeclampsia (n = 31)	Term PE (n = 19)	Preterm PE (n = 12)
Mean maternal age (years)	27.0±7.2	27.9±7.7	28.0±8.1	27.7±7.6
Mean gestational age at delivery (weeks)	39.2±1.2	35.9±4.6*	38.9±1.0	31.2±4.0**
Race, n (%)				
African American	11 (78.6)	25 (80.6)	15 (78.9)	10 (83.3)
Other	3 (21.4)	6 (19.4)	4 (21.1)	2 (16.7)
Mean Body Mass Index (BMI) (lbs/inches^2^)	30.1±8.2‡	30.0±7.4‡	31.7±6.9‡	27.2±7.8‡
Mean Fetal Weight (grams)	3272±467	2676±969*	3239±523	1783±835*
Fetal Gender, n (%)				
Male	5 (35.7)	16 (51.6)	7 (36.8)	9 (75.0)
Female	9 (64.3)	15 (48.4)	12 (63.2)	3 (25.0)

Data expressed as Mean ± SD.

‡ BMI at first prenatal visit with an average gestational age of 18 weeks (controls), 15 weeks (cases), 15 weeks (term PE) or 17 weeks (preterm PE).

*p<0.05 versus Controls, **p<0.0001 versus Term Cases, PE – preeclampsia.

### DNA methylation microarray analysis

**Figure 1:**
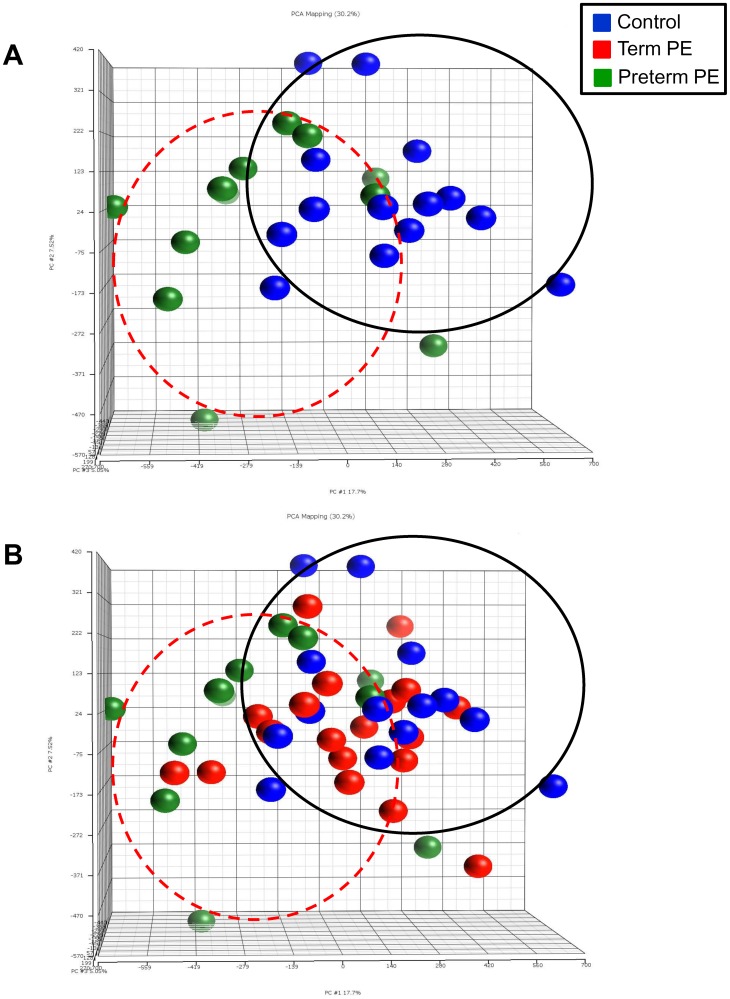
Principle component analysis (PCA) plots of control vs term and preterm preeclamptic placentas. PCA plots show the results of the Illumina Infinium Methylation 450 BeadChip Array comparing (**A**) control placentas to preterm preeclamptic placentas and (**B**) control placentas to term and preterm preeclamptic placentas. The PCA plot was created prior to eliminating gene probes based on p-value or changes in percent methylation. Distinct clustering is seen between normal controls and preterm preeclamptic placentas indicating significant alterations in methylation status in preterm preeclamptic placentas. Each dot represents a single patient sample. Blue dots designate control placentas, green dots designate preterm preeclamptic placentas and red dots designate term preeclamptic placentas.

Using the results from the methylation 450 array, we compared the β values between control, term and preterm PE placentas. Because there are no set standards for analyzing large datasets from methylation arrays, we investigated combinations of Δβ values and significant p-value cutoffs in order to identify a subset of genes for further studies (Table 2). Overall, an initial analysis of the methylation 450 array results with a Step-up (false detection rate) p value cutoff of 0.5, revealed thousands of differentially methylated (hyper- or hypo-methylated) genes between control and PE placentas providing evidence of large methylation effects on PE placentas. Interestingly, the number of differentially methylated probes in term PE vs control was much smaller indicating that placental epigenetic regulation may be more evident with disease severity. Additionally, using the complete data set from the methylation 450 array (prior to limiting gene numbers by p-value or percent methylation differences), principle component analysis (PCA) plots comparing all three groups revealed clustering of the preterm PE samples when compared to controls (Figure 1A) providing some evidence of a larger number of probe sites with methylation differences in the preterm PE group. While no significant clustering is seen with the term PE placentas (Figure 1B), these samples do seem to cluster more closely with the term controls than the preterm PE placentas suggesting that methylation of these samples may be more similar to term controls than those with a more severe form of the disease.

**Table 2:** Number of differentially methylated probes in case versus control placental samples.

Δβ cutoff	0%	0%	5%	5%
Step-up p-value cutoff	0.5	0.05	0.05	0.01
Term + Preterm PE vs Control	179,186	4,316	229	0
Term PE vs Control	209	0	0	0
Preterm PE vs Control	239,433	45,652	3,411	**421**
Preterm PE vs Term PE	159,604	3,422	179	0

Bold number denotes final number of gene probes used for functional analysis.

After eliminating gene probes with Step-up values of greater than 0.05 and focusing on those gene probes with Δβ values greater than 0.05 (5% change in methylation) (Table 2), we found 229 gene probes (representing 154 annotated genes) that were differentially methylated between control and PE (term and preterm) placentas. Of these gene probes, 205 were hypermethylated while 24 were hypomethylated. Interestingly, at this stringency level no genes were differentially methylated between control and term PE placentas. However, we identified 3,411 gene probes (representing 1,448 annotated genes) that were differentially methylated between control and preterm PE placentas. 3,132 gene probes were hypermethylated while 279 were hypomethylated. A total of 179 gene probes (representing 118 annotated genes) were differentially methylated between term PE and preterm PE placentas. 164 gene probes were hypermethylated and 15 were hypomethylated.

**Figure 2:**
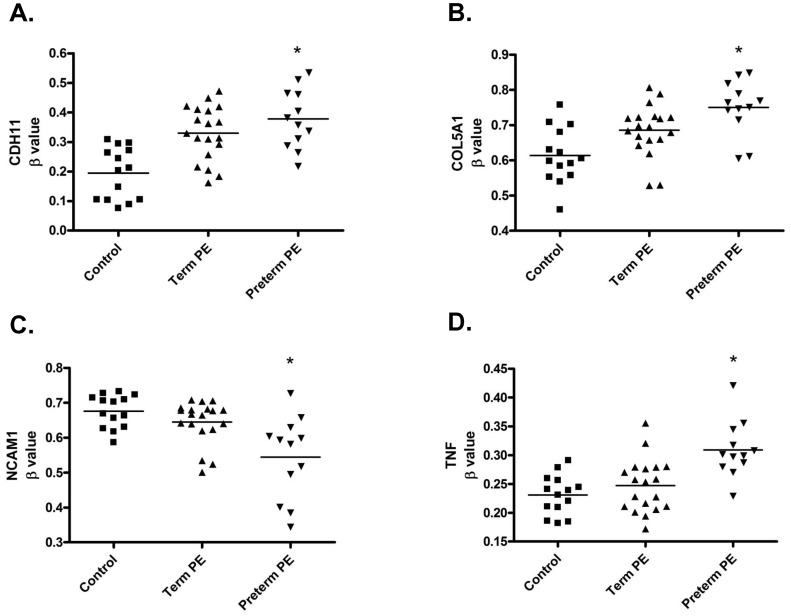
Altered methylation status of four genes of interest in term and preterm preeclamptic placentas. Scatter plots of β values (representing percent methylation) from the Illumina Infinium Methylation 450 BeadChip of array probe CpGs associated with four genes of interest in control versus term and preterm preeclamptic placentas. β values in preterm preeclamptic placentas were significantly altered in (**A**) Cadherin 11 (CDH11) (cg26624576), (**B**) Collagen, type V, alpha 1 (COL5A1) (cg14237069), (C) Neuronal Cell Adhesion Molecule 1 (NCAM1) (cg20857767) and (**D**) Tissue Necrosis Factor (TNF) (cg04425624). Each dot represents a placental sample from each enrolled study patient. Solid bars represent mean of each group. * Statistical significance is based on Step-up (false discovery rate) p-values <0.01.

In order to identify the methylated genes with the highest probability of altering biological function, specifically in the comparison between normal and preterm PE placentas where we saw the greatest number of differentially methylated gene probes, we focused on those that had Δβ values greater than 0.05 with Step-up p values less than 0.01 (Table S2). This resulted in the identification of 421 differentially methylated gene probes (representing 293 annotated genes). 396 were hypermethylated and 25 were hypomethylated. Using DAVID Bioinformatics Resources, we performed a functional annotation analysis of these 293 genes which resulted in the identification of 99 gene annotation clusters with the cadherin and cell adhesion functional clusters being the most significant (Benjamini p-values between 1.2×10^−5^ to 1.7×10^−12^) (Table S3). Similarly, gene ontology analysis of these same genes identified cell adhesion as the most significantly associated biological process (Table S4). 32 of the genes shown to be differentially methylated in preterm PE placentas versus control were found within these functional annotation clusters (Table S5). From this list of 32 genes we chose the four genes with the largest change in methylation status including *CDH11*, *COL5A1*, *NCAM1* and *TNF* to validate methylation changes by an independent method and to further investigate if alterations in methylation resulted in a change in mRNA expression. While the methylation of these four genes of interest was significantly altered in preterm PE placentas, based on a Step-up p value less than 0.05, no changes in methylation were seen in the term PE placentas for *CDH11*, *COL5A1*, *NCAM1* or *TNF* (Table 3, Figure 2).

**Table 3:** Genes associated with the Cell Adhesion functional annotation cluster which showed the highest amounts of differential methylation between control and preterm preeclamptic placentas.

		Control vs Preterm Preeclamptic Placentas			Control vs Term Preeclamptic Placentas		
Gene Name	Probe Location	Δβ Fold Change in Methylation	Percent Change in Methylation	Step-up p-value	Δβ Fold Change in Methylation	Percent Change in Methylation	Step-up p-value
CDH11	TSS1500	1.945	18.3	0.003318	1.698	13.5	0.455744
COL5A1	Gene Body	1.223	13.7	0.005201	1.117	7.2	0.774948
NCAM1	3′UTR	−1.242	−13.2	0.006844	−1.048	−3.1	0.905300
TNF	Promoter Associate First Exon	1.339	7.8	0.005227	1.071	1.6	0.913600

### Pyrosequencing validation of array results

Pyrosequencing assays were done to validate the methylation differences seen in four of the cell adhesion genes (*CDH11*, *COL5A1*, *NCAM1* and *TNF*) identified by the methylation 450 array to be significantly different between control placentas and preterm PE placentas (Table 4). Of these four genes, three of them, *CDH11*, *NCAM1* and *TNF*, showed significant changes in methylation (by pyrosequencing) between control and preterm PE placentas. In agreement with the methylation 450 array, *CDH11* (p = 0.037) and *TNF* (p = 0.030) methylation were significantly increased while *NCAM1* (p = 0.001) methylation was significantly decreased. While showing a similar trend in an increase in methylation between control and preterm PE placentas, percent change in methylation of *COL5A1* did not reach statistical significance (p = 0.129) as was shown in the methylation 450 array. Interestingly, although the methylation 450 array found no significant alterations in methylation in these four genes in the term PE placentas when compared to control (Table 3), pyrosequencing analysis revealed a significant change in methylation status in *CDH11* (p = 0.002) and *NCAM1* (p = 0.002). *COL5A1* (p = 0.144) and *TNF* (p = 0.190) methylation remained not significant (Table 4).

**Table 4:** Pyrosequencing validation of four genes found to have significant differential methylation by the Illumina HumanMethylation 450 array in preterm and term preeclamptic placentas.

	Control vs Preterm Preeclamptic Placentas		Control vs Term Preeclamptic Placentas	
Gene Name	Percent Change in Methylation	p-value	Percent Change in Methylation	p-value
CDH11	6.74	0.037	11.61	0.002
COL5A1	5.06	0.129	4.52	0.144
NCAM1	−6.61	0.001	−5.23	0.002
TNF	3.00	0.030	1.57	0.190

### Methylation status alters mRNA expression

**Figure 3:**
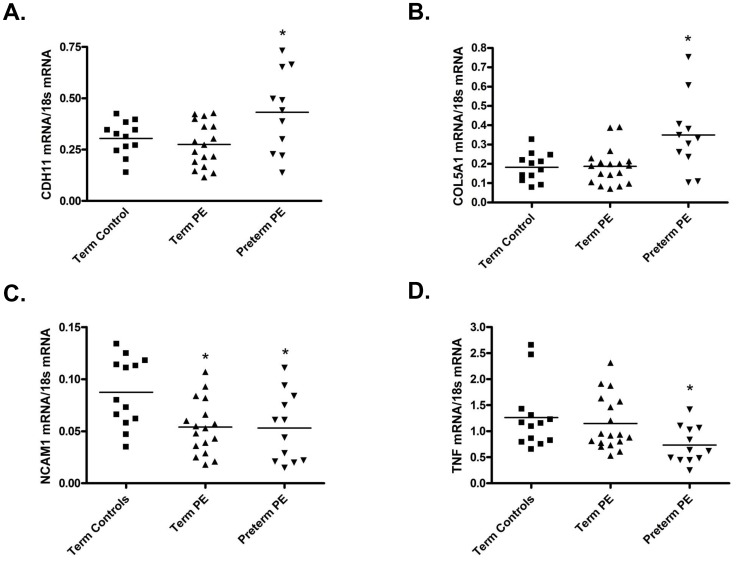
Altered methylation of cell adhesion genes is associated with changes in mRNA expression. Changes in methylation status in term and preterm preeclamptic placentas resulted in altered mRNA expression of (**A**) Cadherin 11 (CDH11), (**B**) Collagen, type V, alpha 1 (COL5A1), (**C**) Neuronal Cell Adhesion Molecule 1 (NCAM1) and (**D**) Tissue Necrosis Factor (TNF). Each dot represents a placental sample from each enrolled study patient. Solid bars represent mean of each group. *p<0.05.

*CDH11*: From the methylation 450 array, of the four genes of interest, *CDH11* showed the largest overall change in methylation status between controls and preterm PE placentas with a Δβ fold change of 1.945 correlating to an 18.3% increase in methylation (p = 0.003318). In term PE placentas, no significant change in *CDH11* methylation status was seen with the stringent statistical requirements of the methylation 450 array (p = 0.455744), however, pyrosequencing did suggest an alteration in methylation (p = 0.002) (Figure 2A, Table 3). Consequently, *CDH11* mRNA expression was significantly increased in preterm PE placentas (p = 0.0143) but not term PE placentas (p = 0.4393) when compared to control (Figure 3A) suggesting that the array results may be more accurate in predicting significant alterations in methylation for this gene.

*COL5A1*: *COL5A1* showed a Δβ fold change of 1.223 correlating to a 13.7% increase in methylation (p = 0.005201) in preterm PE placentas. In term PE placentas, no significant changes in *COL5A1* methylation (p = 0.774948 array, p = 0.144 pyrosequencing) were seen when compared to controls (Figure 2B, Table 3). Similarly to *CDH11*, *COL5A1* mRNA was significantly increased in preterm PE placentas (p = 0.0127) but not term PE placentas (p = 0.8922) when compared to control placentas (Figure 3B).

*NCAM1*: In preterm PE placentas, *NCAM1* showed a Δβ fold change of −1.242 correlating to a 13.2% decrease in methylation (p = 0.006844). In term PE placentas, no significant changes in *NCAM1* methylation were found with the methylation 450 array (p = 0.905300), however, pyrosequencing suggested a significant change in methylation status (p = 0.002) (Figure 2C, Table 3). Consequently, *NCAM1* mRNA was significantly decreased in both preterm (p = 0.0158) and term (p = 0.0042) PE placentas when compared to control (Figure 3C).

*TNF*: In preterm PE placentas, *TNF* showed a Δβ fold change of 1.339 correlating to a 7.8% increase in methylation (p = 0.005227). In term PE placentas, *TNF* methylation status was not significantly altered (p = 0.913600 array, p = 0.190 pyrosequencing) (Figure 2D, Table 3). Hence, *TNF* mRNA was significantly decreased in preterm PE (p = 0.0098) but not term PE (p = 0.5845) placentas when compared to control (Figure 3D).

### Demethylation of first trimester extravillous trophoblasts alters mRNA expression

**Figure 4:**
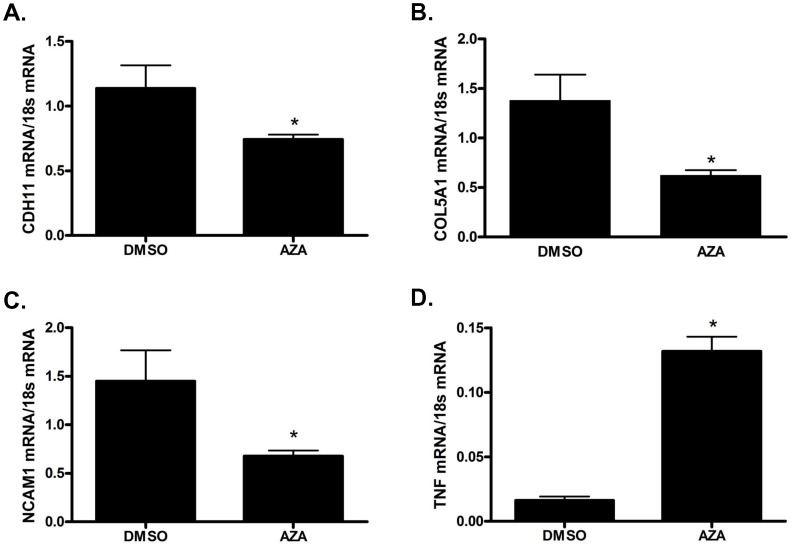
In vitro demethylation of primary first trimester extravillous trophoblast cells results in alterations of mRNA expression. First trimester extravillous trophoblast cells treated with the demethylating agent 5-aza-2′-deoxycytidine (AZA) versus dimethyl sulfoxide (DMSO, vehicle control) showed significant alterations in mRNA expression of (**A**) Cadherin 11 (CDH11), (**B**) Collagen, type V, alpha 1 (COL5A1), (**C**) Neuronal Cell Adhesion Molecule 1 (NCAM1) and (**D**) Tissue Necrosis Factor (TNF). Values are mean ± SEM. n = 6 *p<0.05.

Because we identified significant changes in methylation within the cell adhesion genes *CDH11*, *COL5A1*, *NCAM1* and *TNF* in third trimester preeclamptic placentas, we wanted to investigate if changes in methylation of these specific genes would result in an alteration of mRNA expression in first trimester extravillous trophoblast cells - the cell type whose primary function is invasion of the uterine wall and maternal spiral arteries. First trimester EVT cells treated with the demethylating agent, AZA, resulted in a significant decrease in *CDH11* (p = 0.0087) (Figure 4A), *COL5A1* (p = 0.0043) (Figure 4B) and *NCAM1* mRNA expression (p = 0.0260) (Figure 4C). On the contrary, EVT cells treated with AZA resulted in a significant increase in *TNF* mRNA expression (p = 0.0022) (Figure 4D).

### Placental global methylation is unchanged

In order to determine if there were alterations in global methylation status between control and PE placentas, we performed the LUMA assay (Figure S1). There were no significant changes in percent global methylation between control and PE placentas (controls vs all cases) (p = 0.7158). Furthermore, we investigated if global methylation levels changed in the placenta depending on the severity of preeclampsia. There were no significant differences in percent global methylation between control and term PE placentas (p = 0.3746) or between control and preterm PE placentas (p = 0.6046). Lastly, there were no significant changes in global methylation between term PE and preterm PE placentas (case vs case) (p = 0.2173).

## Discussion

Great strides have been made in understanding the complex genetic contributions to the development of PE; however, investigations into the role of placental epigenetic modifications and their association with placental dysfunction in PE remain in their infancy. While it is generally agreed that alterations in gene methylation lead to changes in gene expression and thus functional alterations within the placenta, a direct link between altered gene-specific DNA methylation and PE-associated placental dysfunction remains to be fully elucidated. In this study, utilizing a large scale genome wide DNA methylation array, we have shown that 1) significant changes in gene methylation status are present in PE placentas when compared to those with uncomplicated term pregnancies, 2) preterm PE placentas have an increased number of differentially methylated genes when compared to control and term PE placentas, 3) those genes with an altered methylation status in preterm PE placentas cluster most significantly in the cell adhesion and cadherin functional groups, 4) altered methylation status of specific cell adhesion genes results in a change in placental mRNA expression, and 5) disruption of methylation in first trimester trophoblasts alters expression of these same genes. These studies demonstrate that differential methylation results in changes in placental mRNA expression suggesting that epigenetic regulation of these genes contributes to placental dysfunction and/or disease development. Furthermore, our results indicate that epigenetic modifications early in pregnancy could have effects on trophoblast function contributing to PE.

Identifying alterations in placental DNA methylation has garnered more interest in the past several years as many believe that epigenetic alterations may play a significant role in the development of adverse obstetrical outcomes, including IUGR and PE. While several studies have sought to investigate changes in DNA methylation in PE placentas, many have focused on global DNA methylation changes [39], [40] or utilized arrays with a small number of gene probes specifically limited to the promoter and/or CpG regions of the gene [20], [21]. We investigated DNA methylation in PE placentas using the Illumina Infinium Human Methylation 450 BeadChip array which not only contains greater than 450,000 gene probes spanning the entire genome but also contains probes outside of promoters or previously identified CpG islands. This format provides a distinct advantage because it is now believed that DNA methylation can occur in areas of the gene outside of known promoter-associated CpG sites and therefore, provides the ability to identify previously unknown sites of methylation. Agreeing with our results, previous studies using a smaller array based format [20], [21] have found significant alterations in DNA methylation in PE placentas when compared to controls. More recently, a study utilizing the methylation 450 array investigated methylation changes in early onset PE compared to preterm controls [22]. While our results are similar in the identification of a large number of differentially methylated genes in the preterm PE placenta, they differ in the usage of preterm controls [22] vs term controls (this study). Additionally, our studies differ in the chosen methylated genes to target and their downstream effects. Overall, the results from our study and this other recent report indicate that placental epigenetic regulation may contribute to the development of PE.

We found a significantly higher number of genes with an altered methylation status in preterm PE placentas as compared to term PE placentas (vs control). Interestingly, in the term PE placentas, methylation status (as shown by array or pyrosequencing) was variable in terms of similarity to controls or preterm PE placentas depending on the specific gene investigated. Notably, women being delivered preterm for a diagnosis of PE are by definition going to have a more severe form of this disease. This is in contrast to women with a diagnosis of PE at term as there is significant variability in the clinical presentation of women with term PE. Hence, it is likely that methylation status of the placenta would vary by disease severity even at term. Understanding that there is more phenotypic variability with PE diagnosed at term than preterm, our findings of more significant methylation changes in preterm PE make biological sense. The different epigenetic profiles shown in preterm PE and term PE placentas suggests that epigenetic modification of placental genes may contribute to the varying disease phenotype of PE. Importantly, altered gene methylation may be mechanistically involved in the pathogenesis of more severe forms (and preterm) of PE. Our findings would suggest that the placental epigenetic modifications observed in our study could occur early in pregnancy contributing to the placental dysfunction that ultimately leads to the development of PE.

A potential limitation of our study is that we cannot ascertain if placental methylation status is altered due to gestational age. While some previous studies have used aged matched spontaneous preterm birth placentas as controls [22], these placentas may inherently contain alterations in DNA methylation similar to those with PE as preterm birth and PE may have some overlapping biologically mechanistic pathways associated with placental dysfunction [41]. While methylation changes based on gestational age do need to be considered as a possible explanation for the increased number of differentially methylated genes observed in preterm PE placentas in our study, it has been previously shown that placental global methylation levels increase with gestational age [42], [43]. Consequently, it would be unlikely that higher amounts of methylation in preterm placentas (compared to term placentas) would be due to an artifact of gestational age. Additionally, the results from our LUMA assay show that global methylation was unchanged between control and PE placentas as well as between term PE and preterm PE placentas. While it is difficult to ascertain from these studies if gestational age has a gene-specific effect, these data would indicate that the observed methylation changes are not due to gestational age alone. A study by Yuen et al. [20] found similar results to ours where early onset PE placentas had 34 differentially methylated gene loci but none were differentially methylated in late onset PE when compared to gestationally aged match controls.

Interestingly, in our study, the genes with the largest changes in DNA methylation in preterm PE placentas clustered into the cadherin and cell adhesion functional groups. The placental genes regulating cell adhesion could significantly contribute to alterations in the migration and invasion of the placental trophoblast cells, a process known to be abnormal in PE placentas [4], [5]. Of the cell adhesion genes we investigated, *CDH11* had the largest alteration in DNA methylation with an 18.3% increase in preterm PE placentas. The *CDH11* hypermethylation identified by the methylation 450 array was further validated by pyrosequencing. It is interesting to note that while *CDH11* methylation levels were not significantly altered in term PE placentas in the methylation 450 array; pyrosequencing did show a significant increase in methylation similar to the preterm PE samples. *CDH11* has been shown to play a role in anchoring trophoblasts to the decidua [44] and terminal differentiation of cytotrophoblast to syncytiotrophoblasts [33], [45] suggesting that *CDH11* contributes to the regulation of trophoblast movement. Highlighting the complexity of epigenetic regulation, in our study, hypermethylation of *CDH11* resulted in an increase in *CDH11* mRNA expression in preterm PE placentas but not in term PE placentas suggesting that the level of methylation may play a role in the downstream regulation of biological function. Interestingly, in agreement with our results, the effects of TGF-β1, a growth factor known to decrease trophoblast invasiveness, are mediated through *CDH11* [33]. Additionally, the expression of *CDH11* is reduced in choriocarcinoma [46], a quick growing trophoblastic cancer, implicating *CDH11* in the regulation of trophoblast proliferation. Furthermore, the observed decrease in *CDH11* expression in demethylated first trimester EVT cells suggest that altered methylation of *CDH11* early in pregnancy has the ability to alter trophoblast function. Therefore, we hypothesize that hypermethylation of *CDH11* resulting in increased *CDH11* mRNA expression could contribute to the decreased trophoblast invasiveness associated with PE.

*COL5A1*, an extracellular matrix (ECM) protein known to be involved in connective tissue disorders, has been identified in placental stromal cells and has been shown to be increased in PE placentas [47]. Similarly, in our study, hypermethylation of *COL5A1* in preterm PE placentas resulted in increased mRNA expression. Of note, *COL5A1* hypermethylation was seen in both the methylation 450 array and the pyrosequencing assay, however, the increase in methylation between control and preterm PE placentas did not reach statistical significance in the pyrosequencing assay. While there is some assumed variability between the two techniques, the overall trend for increased methylation of *COL5A1* and the resulting change in mRNA expression would suggest that this is a mechanistically relevant gene and should not prevent further investigation into the role of *COL5A1* in placental function. However, it is also interesting to note that *COL5A1* methylation and mRNA expression was unchanged in term PE placentas. These results, taken together with our data showing decreased *COL5A1* expression in demethylated first trimester EVT cells, suggests that increased expression of *COL5A1* due to hypermethylation may be associated with alterations in first trimester trophoblast function and, consequently, contributing to disease severity. Although the exact function of *COL5A1* in the PE placenta remains unknown, interactions between the ECM and integrins are known to regulate trophoblast migration [48]. Thus increased *COL5A1* expression due to alterations in gene methylation early in pregnancy may hinder trophoblast motility.

*NCAM1* has been shown to play a role in trophoblast-trophoblast interactions and trophoblast cell adhesion during maternal artery migration [49]. *NCAM1* is decreased in the extravillous trophoblast cells of preeclamptic placentas [50]. Interestingly, in our study, of the four cell adhesion genes investigated, *NCAM1* was the only gene demonstrating a decrease in DNA methylation (validated by pyrosequencing) between controls and preterm PE placentas. In term PE placentas, while *NCAM1* methylation was unchanged in the methylation 450 array, pyrosequencing showed a significant decrease in methylation that was similar to preterm PE samples. Decreased methylation of *NCAM1* resulted in a decrease in mRNA expression in both preterm and term PE placentas suggesting that alterations in methylation result in a change in *NCAM1* gene expression possibly leading to alterations in downstream placental function. Additionally, demethylation of first trimester EVTs resulted in decreased *NCAM1* expression indicating that *NCAM1* may play a specific role in regulating first trimester trophoblast invasion of the maternal spiral arteries and hence may be contributing to the development of preeclampsia from an early time point in pregnancy.

Lastly, *TNF* has been well studied in pregnancy and PE and has been associated with a myriad of bioactivities including immune system activation, cell survival, proliferation, migration and differentiation [51]. Although *TNF* has been shown to inhibit trophoblast migration and invasion [52]–[54], *TNF* has also been shown to stimulate matrix metalloproteinase-9 (MMP-9) [55], [56], a key enzyme in trophoblast invasion. Therefore, the exact role of *TNF* in regulating placental/trophoblast function remains unclear. While there are conflicting reports of altered *TNF* expression in PE placentas [57], [58], in our study hypermethylation of *TNF* resulted in a decrease in *TNF* mRNA expression in preterm PE placentas. Interestingly, there were no changes in *TNF* methylation (array or pyrosequencing) or mRNA expression in term PE placentas providing evidence that epigenetic regulation of *TNF* may be associated with disease severity and may be mechanistically involved in early trophoblast dysfunction leading to a more severe phenotype. Due to the many biological functions attributed to *TNF*, it is difficult to pinpoint the exact effect of decreased *TNF* expression on placental function. However, demethylation resulted in increased *TNF* expression in first trimester EVTs indicating that alteration of *TNF* methylation status early in pregnancy could alter trophoblast function.

While this study cannot determine if differential methylation of *CDH11*, *COL5A1*, *NCAM1* and *TNF* are an end effect of the disease or are causative of the disease, our studies do suggest that these genes are mechanistically involved the development of PE. We acknowledge that additional studies would be needed in order to investigate the effects of these genes (increased or decreased gene expression) on first trimester trophoblast function including EVT invasion and migration.

In the cancer field, it is generally accepted that DNA hypermethylation represses gene transcription through the promoter region of tumor suppressor genes [59]. Therefore, hypermethylation is most often associated with gene silencing/inactivation. Following this paradigm, in our study, when the array gene probe was located in the promoter region of the gene, hypermethylation of *TNF* resulted in decreased gene expression. However, in instances where the gene probe was located in promoter-distal sites (TSS1500 (1.5Kb from TSS) or gene body as designated by Illumina), hypermethylation of *CDH11* and *COL5A1* resulted in an increase in mRNA expression. Furthermore, hypomethylation of *NCAM1* resulted in decreased gene expression when the gene probe was located in the 3′UTR region of the gene. Previous studies have found several examples in both human and mouse where methylation in the gene body is positively associated with gene expression including *Igf2r* [60], *APOE* [61], *Myod1* [62], *IGF2* [63], *BCR-ABL* [64] and *p16 Ex2* [65]. Thus, methylation of a CpG island in a promoter-distal site does not appear to block transcription [66]. Although the field of epigenetics is rapidly evolving, research on epigenetic modifications has been predominantly focused on gene promoters, thus the extent, functional relevance and regulation of tissue-specific DNA methylation at promoter-distal sites remains to be elucidated. However, as DNA methylation and the resulting change in gene expression is known to be regulated in a tissue-specific manner by complex interactions between transcription factors (TF) and enhancers [67], [68] and/or silencers [69], we can hypothesize that hypermethylation of *CDH11* and *COL5A1* may activate the binding of TFs to gene specific enhancers resulting in increased gene expression. On the other hand, *NCAM1* hypomethylation may target TFs to gene specific repressors or block the binding of TFs to enhancers via insulators [70] leading to decreased gene expression. While it is well known that the intricate interactions between DNA methylation and the transcription factors regulating gene expression are tissue and time (development)-specific, ultimately the exact regulation of methylation status at sites away from the promoter remain unknown.

In summary, our results of an altered methylation status in PE placentas suggest that placental epigenetic modifications are associated with the development of PE. Furthermore, the increase in the number of genes methylated in preterm PE placentas points to a correlation between epigenetic regulation and disease severity. Methylation associated changes in *CDH11*, *COL5A1*, *NCAM1* and *TNF* gene expression indicates that alterations in methylation could contribute to functional changes in the placenta. As gene expression in term placentas may not reflect what is occurring during early pregnancy, we investigated the effects of demethylation of first trimester EVTs on gene expression. These studies provide evidence that disruption of methylation in first trimester trophoblasts alters expression of these genes demonstrating that epigenetic modifications early in pregnancy can have effects on trophoblast function contributing to PE. In addition to increasing our understanding of the biological mechanisms contributing to the development of PE, our studies suggest that epigenetic alterations may be targets for future therapeutic interventions in those women with PE.

## Supporting Information

Figure S1 Global methylation levels in control and preeclamptic placentas.
(TIF)

Table S1 Pyrosequencing primers.
(DOCX)

Table S2 Number of differentially methylated probes at specific Δβ and p-value cutoffs in control versus preterm preeclamptic placental samples.
(DOCX)

Table S3 Functional annotation clusters containing differentially methylated genes in control versus preterm preeclamptic placentas.
(DOCX)

Table S4 Gene Ontology (GO) Terms for differentially methylated genes in control versus preterm preeclamptic placentas.
(DOCX)

Table S5 Full list of genes associated with the Cell Adhesion Gene Ontology functional annotation cluster which showed differential methylation between control and preterm preeclamptic placentas.
(DOCX)
