# Supplementary figures and images for: Differential Methylation of Genes Associated with Cell Adhesion in Preeclamptic Placentas
Source: PLoS One. 2014 Jun 25;9(6):e100148. doi: 10.1371/journal.pone.0100148 (PMC4070941; doi:10.1371/journal.pone.0100148)

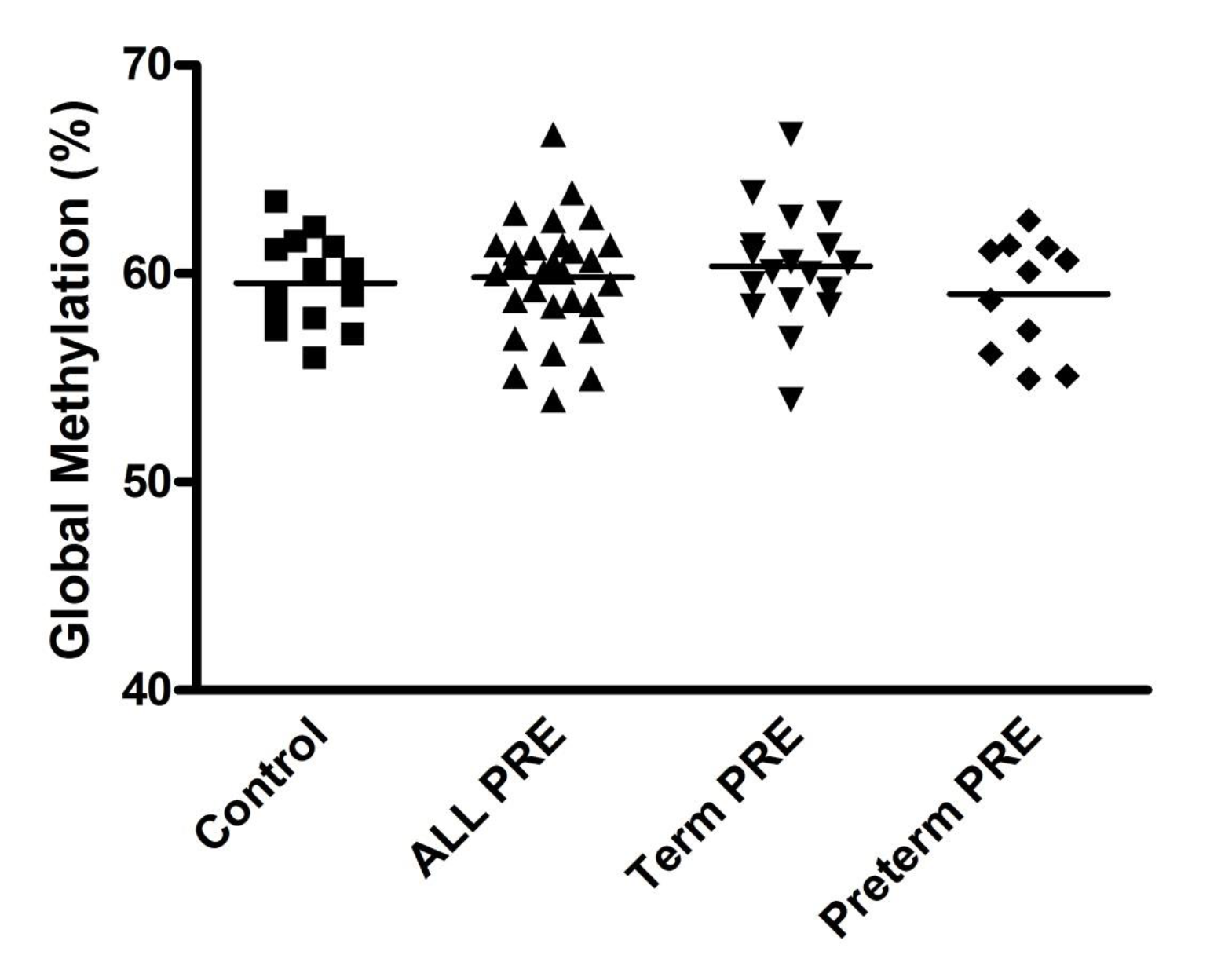

Supplement: Figure S1 — Global methylation levels in control and preeclamptic placentas. (TIF) [file pone.0100148.s001.tif]
