# Supplementary material for: Differential Methylation of Genes Associated with Cell Adhesion in Preeclamptic Placentas
Source: PLoS One. 2014 Jun 25;9(6):e100148. doi: 10.1371/journal.pone.0100148 (PMC4070941; doi:10.1371/journal.pone.0100148)
Supplement: Table S1 — Pyrosequencing primers. (DOCX) [file pone.0100148.s002.docx]

Table S1: Pyrosequencing Primers

| **Gene Name** | **Primer** | **Sequence**  **5’ to 3’** |
| --- | --- | --- |
| CDH11 | F | TTTGGTGGTTTTGGTGGTGAAG |
|  | R-Bio | AACTAAACTCTTCCCAAATCCTAAAT |
|  | Seq | GTTTTGGTGGTGAAGT |
| COL5A1 | F | GAGTGGGGAAGTTTTTTTTTAAAATG |
|  | R-Bio | TTCTTTTAAAACTAACACAAAAAATCCTAA |
|  | Seq | AGTTTTTTTTTAAAATGTAGAAAAT |
| NCAM1 | F | TATTTTTGTGTTTTTTTGGGGGTTAGATTA |
|  | R-Bio | CCCAACTATACAATCTTCTCTACTTCAT |
|  | Seq | GGGGTTAGATTATTTTTTGAT |
| TNF | F-Bio | GGAGAAGAGGTTGAGGAATAAGT |
|  | R | AAACAAATTCTCTTCCTCTCACATACT |
|  | Seq | CCATAAACACTAAAAACATAATC |

F – Forward Primer, R – Reverse Primer, Seq – Sequencing Primer, F-Bio – Biotinylated Forward Primer, R-Bio – Biotinylated Reverse Primer
