# Supplementary material for: Differential Methylation of Genes Associated with Cell Adhesion in Preeclamptic Placentas
Source: PLoS One. 2014 Jun 25;9(6):e100148. doi: 10.1371/journal.pone.0100148 (PMC4070941; doi:10.1371/journal.pone.0100148)
Supplement: Table S2 — Number of differentially methylated probes at specific Δβ and p-value cutoffs in control versus preterm preeclamptic placental samples. (DOCX) [file pone.0100148.s003.docx]

Table S2: Number of differentially methylated probes at specific ΔB and p-value cutoffs in control versus preterm preeclamptic placental samples

|  | ΔB | | | |
| --- | --- | --- | --- | --- |
| Step-up p-value | 0% | 5% | 10% | 15% |
| 0.5 | 239,433 | 25,371 | 2,305 | 251 |
| 0.1 | 72,710 | 7,071 | 998 | 124 |
| 0.05 | 45,652 | 3,411 | 560 | 78 |
| 0.01 | 10,770 | **421** | 79 | 24 |
