# Supplementary material for: Differential Methylation of Genes Associated with Cell Adhesion in Preeclamptic Placentas
Source: PLoS One. 2014 Jun 25;9(6):e100148. doi: 10.1371/journal.pone.0100148 (PMC4070941; doi:10.1371/journal.pone.0100148)
Supplement: Table S3 — Functional annotation clusters containing differentially methylated genes in control versus preterm preeclamptic placentas. (DOCX) [file pone.0100148.s004.docx]

Table S3: Functional Annotation Clusters Containing Differentially Methylated Genes in Control versus Preterm Preeclamptic Placentas

| **Function Annotation Cluster** | **Number of Differentially Methylated Genes in Cluster** | **Benjamini p-value** |
| --- | --- | --- |
| [Cadherin, N-terminal](http://www.ebi.ac.uk/interpro/DisplayIproEntry?ac=IPR013164) | 16 | 1.7E-12 |
| domain:Cadherin 6 | 17 | 4.8E-12 |
| domain:Cadherin 5 | 18 | 1.2E-11 |
| domain:Cadherin 3 | 18 | 2.1E-11 |
| domain:Cadherin 4 | 18 | 2.1E-11 |
| repeat:PXXP 4 | 9 | 2.2E-11 |
| repeat:PXXP 3 | 9 | 2.2E-11 |
| repeat:PXXP 5 | 9 | 2.2E-11 |
| repeat:PXXP 1 | 9 | 2.2E-11 |
| repeat:PXXP 2 | 9 | 2.2E-11 |
| domain:Cadherin 1 | 18 | 2.7E-11 |
| domain:Cadherin 2 | 18 | 2.7E-11 |
| [homophilic cell adhesion](http://www.ebi.ac.uk/ego/DisplayGoTerm?id=GO:0007156) | 19 | 1.0E-9 |
| [cell adhesion](http://us.expasy.org/cgi-bin/get-entries?KW=cell%20adhesion) | 28 | 2.4E-8 |
| [cell-cell adhesion](http://www.ebi.ac.uk/ego/DisplayGoTerm?id=GO:0016337) | 22 | 5.6E-7 |
| [biological adhesion](http://www.ebi.ac.uk/ego/DisplayGoTerm?id=GO:0022610) | 32 | 1.2E-5 |
