# Supplementary material for: Differential Methylation of Genes Associated with Cell Adhesion in Preeclamptic Placentas
Source: PLoS One. 2014 Jun 25;9(6):e100148. doi: 10.1371/journal.pone.0100148 (PMC4070941; doi:10.1371/journal.pone.0100148)
Supplement: Table S4 — Gene Ontology (GO) Terms for differentially methylated genes in control versus preterm preeclamptic placentas. (DOCX) [file pone.0100148.s005.docx]

Table S4: Gene Ontology (GO) Terms for differentially methylated genes in control versus preterm preeclamptic placentas *

| **GO Number** | **GO Term** | **Count** | **Benjamini p-value** |
| --- | --- | --- | --- |
| GO:0007156 | hemophilic cell adhesion | 19 | 1.50E-09 |
| GO:0016337 | cell-cell adhesion | 22 | 8.00E-07 |
| GO:0007155 | cell adhesion | 32 | 2.50E-05 |
| GO:0022610 | biological adhesion | 32 | 1.90E-05 |
| GO:0048731 | system development | 58 | 1.90E-02 |

* Top 5 significant GO Terms out of 47 GO Terms identified by DAVID

Benjamini p-value = Benjamini-Hochberg corrected p-value
