# Supplementary material for: Differential Methylation of Genes Associated with Cell Adhesion in Preeclamptic Placentas
Source: PLoS One. 2014 Jun 25;9(6):e100148. doi: 10.1371/journal.pone.0100148 (PMC4070941; doi:10.1371/journal.pone.0100148)
Supplement: Table S5 — Full list of genes associated with the Cell Adhesion Gene Ontology functional annotation cluster which showed differential methylation between control and preterm preeclamptic placentas. (DOCX) [file pone.0100148.s006.docx]

Table S5: Full list of genes associated with the Cell Adhesion Gene Ontology functional annotation cluster which showed differential methylation between control and preterm preeclamptic placentas

| **GENBANK ACCESSION** | **GENE NAME** |
| --- | --- |
| NM_020693 | [Down syndrome cell adhesion molecule like 1](http://david.abcc.ncifcrf.gov/geneReportFull.jsp?rowids=805989) |
| NM_001797 | [cadherin 11, type 2, OB-cadherin (osteoblast)](http://david.abcc.ncifcrf.gov/geneReportFull.jsp?rowids=817343) |
| NM_022124 | [cadherin-like 23](http://david.abcc.ncifcrf.gov/geneReportFull.jsp?rowids=803844) |
| NM_001741,  NM_001033952, NM_001033953 | [calcitonin-related polypeptide alpha](http://david.abcc.ncifcrf.gov/geneReportFull.jsp?rowids=774578) |
| NM_000093 | [collagen, type V, alpha 1](http://david.abcc.ncifcrf.gov/geneReportFull.jsp?rowids=780010) |
| NM_014141 | [contactin associated protein-like 2](http://david.abcc.ncifcrf.gov/geneReportFull.jsp?rowids=806583) |
| NM_001338 | [coxsackie virus and adenovirus receptor pseudogene 2; coxsackie virus and adenovirus receptor](http://david.abcc.ncifcrf.gov/geneReportFull.jsp?rowids=811214) |
| NM_018891,  NM_005562 | [laminin, gamma 2](http://david.abcc.ncifcrf.gov/geneReportFull.jsp?rowids=801278) |
| NM_000615,  NM_181351 | [neural cell adhesion molecule 1](http://david.abcc.ncifcrf.gov/geneReportFull.jsp?rowids=774998) |
| NM_138970,  NM_001105250,  NM_004796 | [neurexin 3](http://david.abcc.ncifcrf.gov/geneReportFull.jsp?rowids=814893) |
| NM_001048209, NM_001144059,  NM_016522,  NM_001144058 | [neurotrimin](http://david.abcc.ncifcrf.gov/geneReportFull.jsp?rowids=826241) |
| NM_130855,  NM_130853,  NM_130854,  NM_002850 | [protein tyrosine phosphatase, receptor type, S](http://david.abcc.ncifcrf.gov/geneReportFull.jsp?rowids=817489) |
| NM_018902 | [protocadherin alpha 11](http://david.abcc.ncifcrf.gov/geneReportFull.jsp?rowids=819185) |
| NM_018903 | [protocadherin alpha 12](http://david.abcc.ncifcrf.gov/geneReportFull.jsp?rowids=788726) |
| NM_018901,  NM_031882 | [protocadherin alpha 13; protocadherin alpha 10; protocadherin alpha subfamily C, 1; protocadherin alpha subfamily C, 2](http://david.abcc.ncifcrf.gov/geneReportFull.jsp?rowids=800711) |
| NM_018900,  NM_018907,  NM_031411 | [protocadherin alpha 1; protocadherin alpha 4](http://david.abcc.ncifcrf.gov/geneReportFull.jsp?rowids=804361) |
| NM_018905 | [protocadherin alpha 2](http://david.abcc.ncifcrf.gov/geneReportFull.jsp?rowids=825014) |
| NM_018906 | [protocadherin alpha 3](http://david.abcc.ncifcrf.gov/geneReportFull.jsp?rowids=797820) |
| NM_018908 | [protocadherin alpha 5](http://david.abcc.ncifcrf.gov/geneReportFull.jsp?rowids=824691) |
| NM_018910,  NM_031852 | [protocadherin alpha 7](http://david.abcc.ncifcrf.gov/geneReportFull.jsp?rowids=798385) |
| NM_031849,  NM_018909,  NM_018911 | [protocadherin alpha 8; protocadherin alpha 6](http://david.abcc.ncifcrf.gov/geneReportFull.jsp?rowids=824716) |
| NM_018940 | [protocadherin beta 7](http://david.abcc.ncifcrf.gov/geneReportFull.jsp?rowids=792367) |
| NM_018912 | [protocadherin gamma subfamily A, 1](http://david.abcc.ncifcrf.gov/geneReportFull.jsp?rowids=789016) |
| NM_018914,  NM_032092 | [protocadherin gamma subfamily A, 11](http://david.abcc.ncifcrf.gov/geneReportFull.jsp?rowids=783483) |
| NM_018917 | [protocadherin gamma subfamily A, 4](http://david.abcc.ncifcrf.gov/geneReportFull.jsp?rowids=821416) |
| NM_018921 | [protocadherin gamma subfamily A, 9](http://david.abcc.ncifcrf.gov/geneReportFull.jsp?rowids=774317) |
| NM_018922 | [protocadherin gamma subfamily B, 1](http://david.abcc.ncifcrf.gov/geneReportFull.jsp?rowids=822698) |
| NM_018926 | [protocadherin gamma subfamily B, 6](http://david.abcc.ncifcrf.gov/geneReportFull.jsp?rowids=789045) |
| NM_001099401, NM_001099400,  NM_003919 | [sarcoglycan, epsilon](http://david.abcc.ncifcrf.gov/geneReportFull.jsp?rowids=789102) |
| NM_014441 | [sialic acid binding Ig-like lectin 9](http://david.abcc.ncifcrf.gov/geneReportFull.jsp?rowids=819576) |
| NM_019105 | [tenascin XB; tenascin XA pseudogene](http://david.abcc.ncifcrf.gov/geneReportFull.jsp?rowids=790976) |
| NM_000594 | [tumor necrosis factor (TNF superfamily, member 2)](http://david.abcc.ncifcrf.gov/geneReportFull.jsp?rowids=813881) |
